# Reproductive Acclimation to Increased Water Temperature in a Tropical Reef Fish

**DOI:** 10.1371/journal.pone.0097223

**Published:** 2014-05-13

**Authors:** Jennifer M. Donelson, Mark I. McCormick, David J. Booth, Philip L. Munday

**Affiliations:** 1 School of the Environment, University of Technology, Sydney, Broadway, New South Wales, Australia; 2 School of Marine and Tropical Biology, and ARC Centre of Excellence for Coral Reef Studies, James Cook University, Townsville, Queensland, Australia; University of California- Santa Barbara, United States of America

## Abstract

Understanding the capacity of organisms to cope with projected global warming through acclimation and adaptation is critical to predicting their likely future persistence. While recent research has shown that developmental acclimation of metabolic attributes to ocean warming is possible, our understanding of the plasticity of key fitness-associated traits, such as reproductive performance, is lacking. We show that while the reproductive ability of a tropical reef fish is highly sensitive to increases in water temperature, reproductive capacity at +1.5°C above present-day was improved to match fish maintained at present-day temperatures when fish complete their development at the higher temperature. However, reproductive acclimation was not observed in fish reared at +3.0°C warmer than present-day, suggesting limitations to the acclimation possible within one generation. Surprisingly, the improvements seen in reproduction were not predicted by the oxygen- and capacity-limited thermal tolerance hypothesis. Specifically, pairs reared at +1.5°C, which showed the greatest capacity for reproductive acclimation, exhibited no acclimation of metabolic attributes. Conversely, pairs reared at +3.0°C, which exhibited acclimation in resting metabolic rate, demonstrated little capacity for reproductive acclimation. Our study suggests that understanding the acclimation capacity of reproductive performance will be critically important to predicting the impacts of climate change on biological systems.

## Introduction

Successful reproduction is paramount to individual fitness and population replenishment. As population size depends on the balance between population increase (births plus immigrants) and decrease (deaths plus emigrants), any sustained reduction in reproductive rate could have serious consequences for population persistence. Global warming has already caused significant changes to biological communities including advancement in the timing of reproductive events and shifts in the geographic distributions of many taxa [1]–[3]. Changes in the timing of reproduction are expected because reproduction generally only occurs over a small subset of the range of temperatures in which individuals persist, due to the energetic costs involved [4]–[5]. While shifts in reproductive timing to coincide with changing thermal conditions could be adaptive, they may also cause a mismatch in food availability for offspring when all trophic levels are not equally affected by the changing environment [6]–[7]. Consequently, populations will also need to adjust to rising temperatures over coming decades, either through phenotypic plasticity (acclimation) or genetic adaptation [8]–[9]. Modelling of future warming scenarios suggest that projected warming may be too fast for adaptation to keep pace in species with long generation times [10]–[11]. So far investigations into the acclimation ability of reproductive traits other than the timing of breeding has suggested limited capacity to shift in short time frames [11]–[12] and consequently reproduction could be the greatest challenge for some species in a warmer world [12].

Ecotherms are expected to be especially sensitive to environmental warming due to their lack of thermal regulation. This lack of temperature regulation means that cellular function and performance is tightly linked to environmental temperature [13]–[15], and consequently the behaviour and distribution of many ectotherms is controlled by temperature [16]. For aquatic ectotherms, recent research has suggested that key physiological traits related to oxygen transport are responsible for their response to rising temperatures and likelihood of persistence as the climate changes [15]–[19]. The oxygen- and capacity-limited thermal tolerance hypothesis (OCLTT) proposes that as temperature rises above the thermal optimum, the capacity for oxygen transport or aerobic capacity is reduced [17], [20]. This reduced aerobic capacity is caused by the inability of the circulatory and ventilatory systems to keep pace with the oxygen demands. It is expected that this constraint on aerobic performance will then influence all higher-level function including behaviour, growth, swimming ability and reproduction [18], [21], ultimately leading to population declines.

Reproductive performance of aquatic ectotherms may also be influenced by environmental temperature independent of oxygen transport. For example, temperature can directly affect reproductive performance through its influence on sexual development and the timing of maturation [22]–[23], and through the thermal sensitivity of reproductive hormones and enzymes [23]–[24]. Temperature can directly affect reproductive processes by promoting or inhibiting hormone synthesis and action within the hypothalamo–pituitary–gonadal (HPG) axis, and though alteration of hormone structure, both of which are critical to gamete development [23]. Experimental investigations have shown strong upper thermal thresholds to reproductive activity [4], [25]–[26] and near this upper threshold the number and quality of offspring is reduced [25]. This suggests that plasticity of either the seasonal timing of reproduction, or the thermal optimum/threshold for reproduction is likely to be key for population and species persistence with future warming.

When fish are reared from hatching at elevated temperatures, thermal acclimation that would otherwise not occur at later life stages [27], has been observed [28]–[30]. The performance traits of individuals that develop at these warmer temperatures is greater than other individuals from the same cohort that have been reared at current-day temperatures and then tested at elevated temperatures later in life (see also [31]). This is called developmental thermal acclimation. However, not all performance traits will respond exactly the same to elevated temperature and in some cases tradeoffs can exist [28]. In addition, the capacity to undergo developmental acclimation is likely to vary substantially between marine fish species due to diversity in life histories. Studies also show that the thermal environment experienced by parents can affect the performance of offspring in that environment in the next generation [32]–[33]. However, studies of developmental thermal acclimation and parental effects in relation to global warming have so far only focused on growth, swimming ability and metabolic attributes [28], [30]–[33]. It is unknown whether acclimation of reproductive performance is possible with development in elevated thermal conditions. If reproductive acclimation is possible the OCLTT hypothesis predicts that that plasticity of reproductive performance will correlate to changes in aerobic metabolism.

In this study we tested the potential for improved reproductive performance of a common coral reef fish through developmental acclimation to elevated ocean temperatures predicted with global warming. Because the same fish had previously been tested for the potential for acclimation of aerobic metabolism [28]–[29] to elevated temperatures, we were also able to determine if there was a correlation between acclimation in reproductive and aerobic performance, as predicted by the OCLTT hypothesis. We reared fish from shortly after hatching to maturity in present-day (+0.0°C) and elevated temperatures (+1.5 and +3.0°C), on a seasonally fluctuating cycle, to determine if continuous exposure to elevated temperatures induced an acclimation response in a broad range of reproductive traits, including: eggs size, fecundity, reproductive output, size at hatching and body condition of offspring. Additionally, the offspring of control and developmentally acclimated adult fish were reared for 30 days post-hatching under all three treatment temperatures (fully-crossed) to determine if parental effects on offspring attributes at hatching, influence growth and development of offspring in their early life. Finally, the hypothesis that acclimation ability of reproductive performance would match that of aerobic metabolism was tested by comparing results attained in this study with previous results on aerobic performance. The same fish utilized in this study have been shown to developmentally acclimate resting metabolic rate at +3.0°C conditions, but exhibit no improvement in aerobic scope and no acclimation of any metabolic trait for fish that develop in +1.5°C conditions [29]. Consequently, we were able to explore whether acclimation of reproductive traits mirrors the observed trends in metabolic traits.

## Materials and Methods

### Ethics Statement

F_0_ adults were collected from the Great Barrier Reef, Australia under a permit obtained from the Great Barrier Reef Marine Park Authority (G03/3871.1). Individuals were caught using hand nets following a clove oil and alcohol solution being sprayed into the water surrounding fish to anaesthetize before capture. All studies were conducted in accordance with Australian Code of Practice for the Care and Use of Animals for Scientific Purposes 7th Edition, 2004 and in compliance with the Queensland Animal Care and Protection Act, 2001. Specifically, this project was completed under JCU Ethics A1233 and A1415. All sampled fish were euthanised with an overdose of clove oil and seawater solution.

### Experimental Design

The species used in this study was the coral reef damselfish *Acanthochromis polyacanthus*, a widespread Indo Pacific species (15°N–26°S and 116°E–169°E) that broods its young [34]. Fish were collected from the Palm Island region (18° 37′S, 146° 30′E) of the central Great Barrier Reef, which experiences a mean annual temperature range of 23.2°C to 28.5°C. Average sea surface temperatures in the Great Barrier Reef, Australia, are predicted to increase by up to 3°C by 2100 due to global warming [35]–[36]. Consequently, temperature treatments of +1.5°C or +3.0°C were chosen to reflect moderate and more extreme warming by 2100.

Nine established pairs of *A. polyacanthus* were collected for broodstock during July to August 2007 and maintained in 60 l aquariums inside an environmentally-controlled facility at James Cook University, Townsville, Australia. Pairs were maintained at the mean present-day ocean temperature (on a seasonally fluctuating cycle) for the collection location and provided with the average food consumed by wild pairs [25]. During the austral summer 2007–2008, breeding bouts from 8 adult pairs were used for the current study. Offspring from these pairs were kept with their parents for 30 days post-hatching to match field observations [37]. At this time the offspring (F_1_ individuals) from each clutch were divided into 3 groups for rearing in 3 seasonally-cycling temperatures regimes; splitting clutches in this way ensured that each experimental treatment contained similar genetic diversity. One treatment group was kept at the present-day average temperature (seasonal cycle, winter minimum 22.4°C) at the collection location (+0.0°C), while the other two groups were gradually adjusted to, and reared at, two higher temperature treatments: either +1.5°C or +3.0°C (seasonal cycle, winter minimum 23.9°C and 25.4°C respectively; see Donelson et al. 2011 for more detail). Temperature was kept within ±0.2°C of the desired daily temperature for each treatment. Temperatures at the collection location have naturally fluctuated between 0.2–2.5°C in a single day, but on average vary only 0.45°C daily (JCU/AIMS weather station 1999–2008). Sibling fish were kept in groups of 6 in 40 l aquaria for 1 year after hatching, at which time density was reduced to pairs. At 1.5 years fish were reorganised in non-sibling pairs for breeding at 2 years when maturity was reached (See [28] for more details). Aerobic performance of these fish was tested at the end of the breeding season at 2 years of age, as described in Donelson and Munday 2012 [29].

### Reproduction and Offspring Characteristics

During the austral summer 2009–2010 nesting sites of F_1_ breeding pairs were checked daily at 09∶00 for the presence of eggs. When a clutch was discovered an underwater photograph was taken for estimation of the number of eggs laid as well as a sample of 10 eggs from random locations within the clutch to determine egg size (to nearest 0.01 mm, see [25] for methods). The values for mean egg size and number per clutch were used to calculate an estimate of reproductive output (mean egg area x clutch size). Following the observation of a clutch, tanks were checked again daily at 11∶00 for the presence of hatched offspring. Directly after hatching, a sample of 20 offspring were removed and euthanized to subsequently determine offspring characteristics with image analysis (standard length to nearest 0.01 mm: SL, weight to nearest mg: W and yolk area to nearest 0.01 mm: YA; see [38]).

### Adult Attributes

At the conclusion of the breeding season (April 2010) all mature F_1_ fish were euthanised for morphological measurements. SL (to nearest 0.01 mm), W (wet to 0.01 g) and liver weight (to 0.001 g) were recorded. Hepato-somatic index (HSI) was then calculated as liver weight as a percentage of W. HSI serves as a proxy for the energy status of individuals.

### Juvenile Performance

To test differences in performance of offspring produced by F_1_ pairs maintained at the three developmental temperature treatments, an additional 60 newly hatched offspring from the pairs were haphazardly sampled from each clutch and transferred into individual 2 l plastic aquaria and supplied with a constant flow of seawater. These 60 fish were divided evenly among the 3 treatments groups (n = 20). Treatments were the present-day summer average temperature at the collection location (28.5°C), +1.5°C (30.0°C) and +3.0°C (31.5°C) above the summer average. Fish were gradually adjusted to the various treatment temperatures over 4–5 hours. Feeding rates of juveniles followed [26] ‘high food’ treatment. Briefly, juveniles were fed *Artemia nauplii* at a concentration of 2 individuals ml^−1^ day^−1^ from day 1–7 post-hatching, subsequently from day 7–15 *Artemia* at 1 individual ml^−1^ and approximately 2 mg of INVE Aquaculture Nutrition 2/4 NRD pellets day^−1^, and from day 15–30 juveniles were fed approximately 5 mg of 2/4 NRD. Aquariums were checked daily at 09∶00 h, and deaths within the previous 24 h period were recorded. Survival was >85% within all parental treatments groups at all juvenile rearing temperatures. Half of the individuals still alive were sampled at day 15 of the experiment, and all the remaining individuals were sampled at day 30 post-hatching. Fish were euthanised and measured on the day of sampling prior to preservation; SL was measured to the nearest 0.01 mm and W (wet) to nearest mg.

### Statistics

All offspring characteristics (SL, W, YA and Fulton’s K condition index) and egg area were tested with nested ANOVAs; F_1_ developmental treatment temperature (fixed factor) and breeding pairs nested within the developmental treatment (random factor). Other reproductive characteristics, including the number of eggs produced in a clutch and reproductive output were tested with a one-way ANOVA to determine the effect of developmental treatment temperature. The proportion of reproductively active pairs was examined with a 2×3 chi squared test (2 categories breeding or non-breeding and 3 temperature treatments). The number of clutches each pair produced was explored with a Kruskal-Wallis ANOVA by ranks. Reproductive acclimation of an attribute was considered to occur if the elevated treatment group was not significantly different from the +0.0°C control.

Adult characteristics (SL, W and HSI) measured at the conclusion of the summer season were tested with one-way ANOVAs. All *post-hoc* testing was completed with Tukey’s HSD and significance was determined when p<0.05.

The characteristics (SL, W and Fulton’s K condition) of F_2_ juveniles reared for 15 and 30 days in a fully orthogonal cross were tested with a combined nested and factorial ANOVA; parental treatment (fixed factor), pairs nested within the parental treatment (random factor) and juvenile development temperate (fixed factor). In all the above tests the data met the required statistical assumptions.

## Results

### Reproductive and Offspring Characteristics

The proportion of F_1_ fish breeding within each temperature treatment declined with increasing temperatures; 64% of fish reproduced in +0.0°C, 54% in +1.5°C and 36% in +3.0°C, however this trend was not significant (*X* ^2^ = 1.94, df = 2, p = 0.379). The total number of clutches produced by pairs in the treatments declined with increasing temperature: +0.0°C = 14 clutches, +1.5°C = 10 clutches and +3.0°C = 4 clutches and this difference was due to the pairs at the warmest temperature producing fewer clutches per pair (H_2,18_ = 6.88, p = 0.03). There was no difference in the onset of reproduction, with all treatment groups commencing breeding in late October; however pairs in the +3.0°C treatment ceased breeding in late December (8 weeks after reaching summer temperatures) while +0.0°C and +1.5°C pairs continued to breed until February.

**Figure 1:**
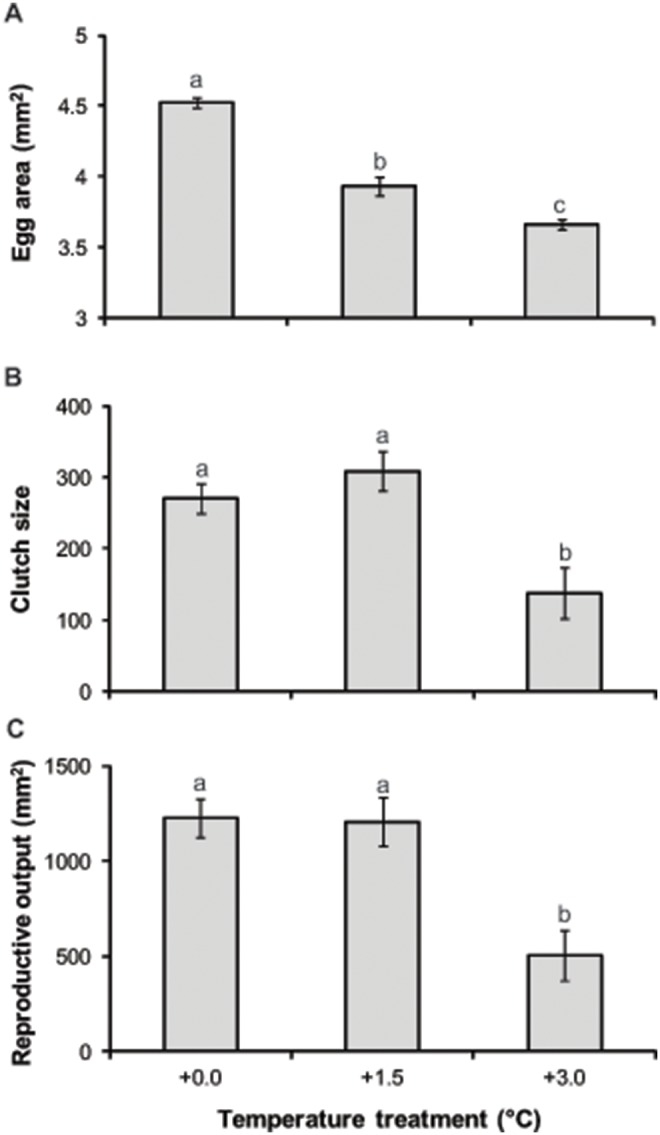
Reproductive performance of treatment groups. Mean (a) egg area, (b) clutch size and (c) reproductive output (mean egg area × clutch size) of *Acanthochromis polyacanthus* pairs maintained under the three temperature treatments. Values are mean ± SE. Letters represent significant differences between treatments (p<0.05).

F_1_ breeding pairs reared at higher temperatures produced smaller eggs than control pairs (Fig. 1a; F_2,15_ = 8.19, p = 0.004). The mean size of eggs at +1.5°C was 13% smaller and at +3.0°C 19% smaller than those produced by +0.0°C pairs. Pairs from the warmest +3.0°C treatment also produced significantly fewer eggs per clutch (Fig. 1b; F_2,15_ = 7.79, p = 0.005) and exhibited lower reproductive output (Fig. 1c; F_2,15_ = 8.71, p = 0.003) compared to fish maintained at the present-day +0.0°C temperature and +1.5°C. Both clutch size and reproductive output were at least 50% lower at +3.0°C compared with +0.0°C and +1.5°C, however no difference in reproductive output was observed between +0.0 and +1.5°C.

**Figure 2:**
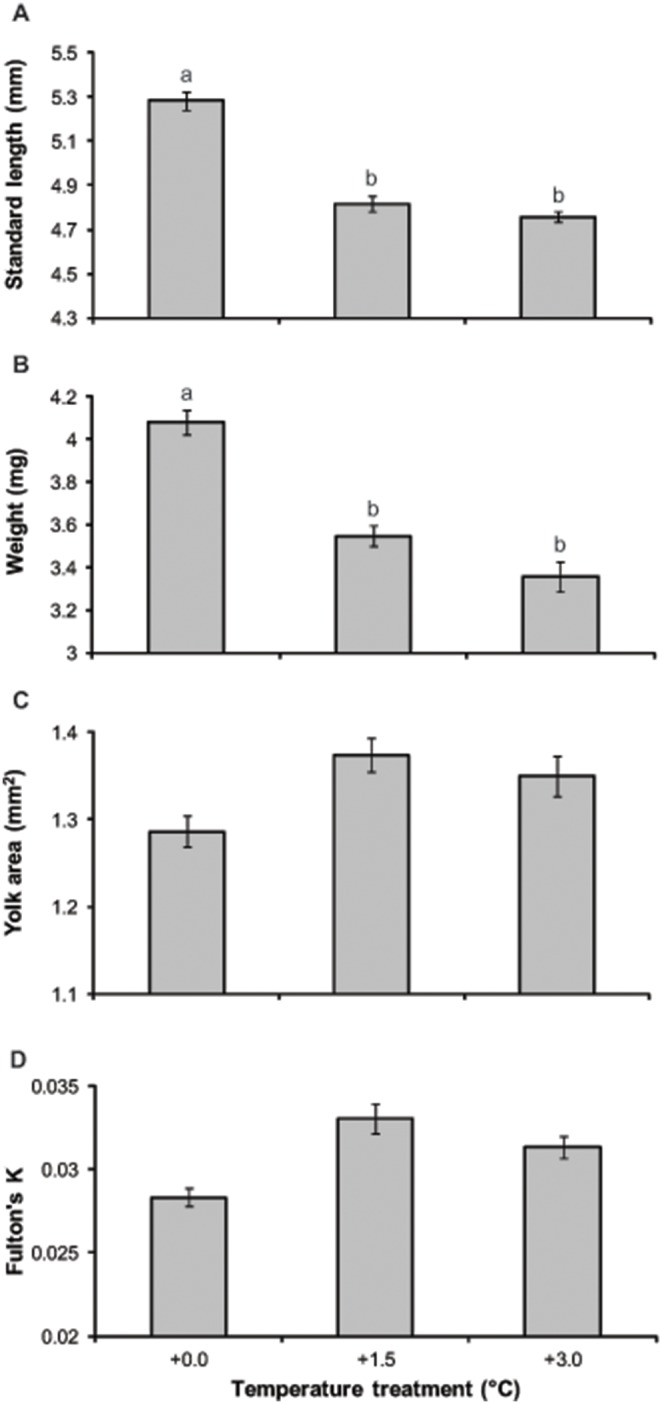
Offspring characteristics at hatching depending on parental treatment groups. Mean (a) standard length, (b) weight, (c) yolk area and (d) Fulton’s K condition of *Acanthochromis polyacanthus* offspring that resulted from pairs maintained under the three temperature treatments. Values are mean ± SE. Letters represent significant differences between treatments (p<0.05).

At hatching, offspring produced by parents that had been reared in elevated temperature treatments (both +1.5°C and +3.0°C) were shorter (Fig. 2a; F_2,15_ = 4.27, p = 0.034) and lighter (Fig. 2b; F_2,15_ = 4.56, p = 0.028), but possessed the same amount of yolk (Fig. 2c; F_2,15_ = 0.33, p>0.05) and physical condition (Fig. 2d; F_2,15_ = 1.01, p>0.05) compared to the present-day +0.0°C control. However, not all breeding pairs within a treatment responded the same to water temperature. In the case of egg area and all newly hatched offspring attributes measured (SL, W, YA and Fulton’s K) there were significant differences among breeding pairs within temperature treatments (SL, F_15,329_ = 33.84, p<0.001; W, F_15,329_ = 12.66, p<0.001; YA, F_15,330_ = 30.81, p<0.001; Fulton’s K, F_15,328_ = 20.41, p<0.001).

### Adult Attributes

Adult F_1_ fish that developed at various temperature treatments possessed significantly different physical characteristics. Adult F_1_ fish maintained in the warmest temperature treatment (+3.0°C) were significantly shorter, by 4 mm on average, and lighter, by 4 g on average, than the adults of the other temperature treatments (Table 1, p<0.05). In the case of HSI, fish maintained at +0.0°C had significantly lower values (p<0.05) than fish maintained at +1.5C and +3C.

**Table 1:** Mean standard length (SL), weight and hepato-somatic index (HSI) ± SE of F_1_ adults reared from near hatching in three temperature treatments.

Treatment	SL	Weight	HSI
+0.0°C	78.82±1.23	21.55±1.14	0.93±0.05
+1.5°C	79.13±1.09	21.10±0.91	1.09±0.05
+3.0°C	74.79±1.18	17.33±1.00	1.32±0.05
ANOVA	F_2,69_ = 4.73 p<0.01	F_2,69_ = 4.22 p = 0.019	F_2,69_ = 11.99 p<0.010

### Juvenile Performance

**Figure 3:**
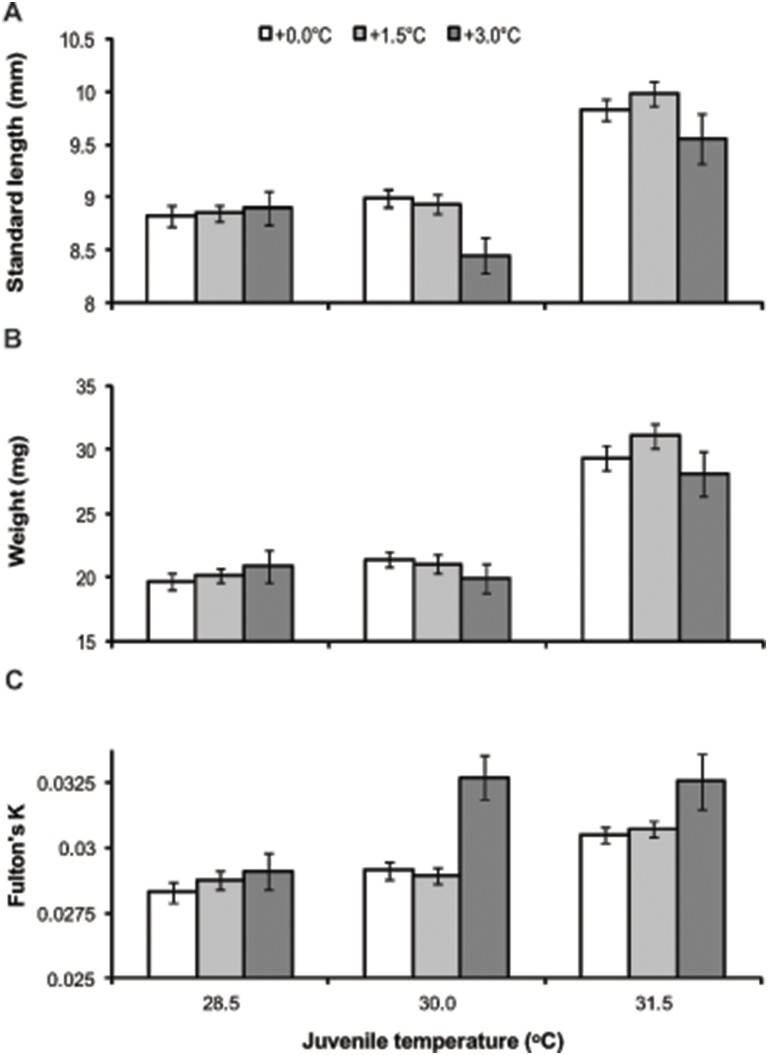
Offspring performance at 15 days post-hatching across all summer treatment temperatures. Effect of parental treatment and juvenile rearing conditions on the standard length (SL), weight (W) and Fulton’s K condition index at 15 days post-hatching of *Acanthochromis polyacanthus*. Values are means ± SE.

**Figure 4:**
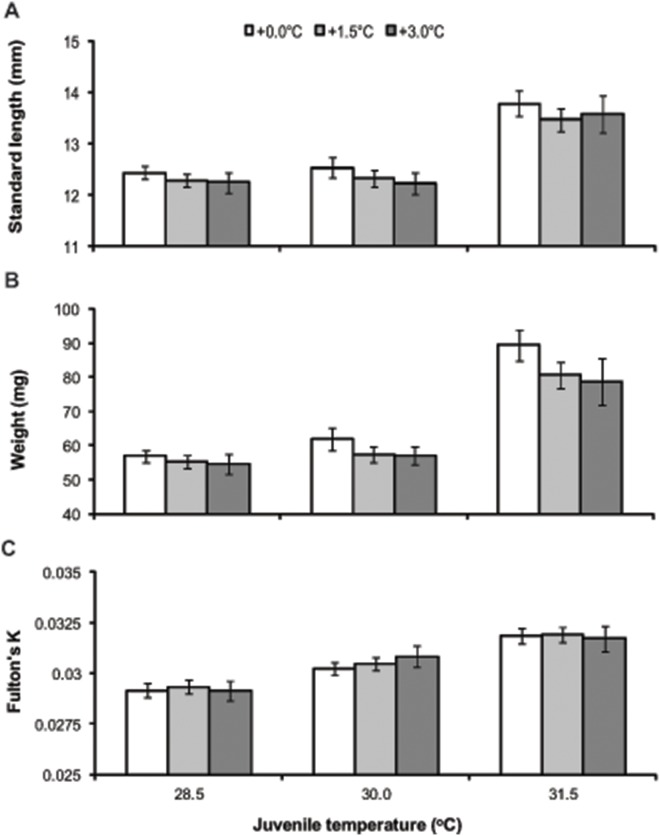
Offspring performance at 30 days post-hatching across all summer treatment temperatures. Effect of parental treatment and juvenile rearing conditions on the standard length (SL), weight (W) and Fulton’s K condition index at 30 days post-hatching of *Acanthochromis polyacanthus*. Values are means ± SE.

Juvenile growth and body condition were strongly influenced by juvenile rearing temperature at both 15 and 30 days post-hatching (Fig. 3 & 4; Juvenile treatment 15 days = SL, F_2,355_ = 45.31, p<0.001; W, F_2,355_ = 59.35, p<0.001; Fulton’s K, F_2,355_ = 10.32, p<0.001; Juvenile treatment 30 days = SL, F_2,336_ = 34.77, p<0.001; W, F_2,336_ = 56.24, p<0.001; Fulton’s K, F_2,336_ = 40.24, p<0.001). However, offspring produced by parents maintained in elevated temperature conditions were able to compensate for their smaller size at hatching by accelerated growth within the first 15 days of life, regardless of juvenile rearing temperature (Fig. 3; Parental treatment 15 days = SL, F_2,11_ = 0.58, p>0.05 and W, F_2,11_ = 0.14, p>0.05). As observed for attributes at hatching, juveniles from different breeding pairs performed differently within a temperature treatment at both 15 and 30 days post-hatching, except for Fulton’s K condition at 15 days (Pair (Parental treatment) 15 days = SL, F_11,355_ = 3.21, p = 0.007; W, F_11,355_ = 2.90, p = 0.013; Pair (Parental treatment) 30 days = SL, F_11,336_ = 7.49, p<0.001; W, F_11,336_ = 7.59, p<0.001; Fulton’s K, F_11,336_ = 8.02, p<0.001). However, an interaction between juvenile rearing temperature and pairs within temperature treatments was also found (Juvenile treatment*Pair (Parental treatment) 15 days = SL, F_26,355_ = 2.24, p<0.001; W, F_26,355_ = 2.62, p<0.001; Fulton’s K, F_26,355_ = 2.73, p<0.001; Juvenile treatment*Pair (Parental treatment) 30 days = SL, F_26,336_ = 1.71, p = 0.018; W, F_26,336_ = 1.74, p = 0.015). This significant interaction was caused by some pairs within both the +0.0°C and +1.5°C treatment producing offspring that grew significantly longer and heavier in warm conditions (30.0°C and 31.5°C) compared to offspring produced by other pairs within the same parental treatment.

## Discussion

Reproductive performance of ectothermic species may be negatively impacted by future warming. However, past research has generally focused on the effects of short-term temperature increases to already mature animals, thus the capacity for reproductive acclimation when warmer conditions are experienced throughout development has not been established. We found evidence for acclimation of some reproductive traits when individuals of a tropical reef fish developed in ocean temperatures 1.5°C greater than present-day for their entire life. However, this was not the case when individuals experienced +3.0°C warmer conditions, suggesting limitations to thermal acclimation. Surprisingly, the improvements in reproductive ability in +1.5°C fish did not match the patterns of metabolic acclimation we observed in the same fish [29]. This suggests that the effects of elevated temperature on reproduction are independent of the effects on metabolism and cannot be explained by the oxygen- and capacity-limited thermal tolerance hypothesis (OCLTT; [17], [20]). Our results show that some species may have the ability for acclimation to warmer environmental conditions when they experience elevated temperature from early life and this improvement may have important consequences for future generations. Our results also indicate that studies that examine the short-term impacts of temperature on populations may overestimate the negative temperature effects by not allowing for developmental acclimation.

In the previous generation of the fish used in this study, when adult breeding pairs collected from the wild were placed under the same elevated temperature conditions as used here, reductions in the proportion of pairs reproducing were observed together with reductions in reproductive output, the size of eggs and the number of eggs in each clutch [25]. In the current F_1_ generation, when fish were reared at +1.5°C temperatures, we saw improvements in the proportion of pairs reproducing, the number of eggs per clutch and the reproductive output, but not the size of eggs. These improvements suggest that acclimation of reproductive ability is possible when generations experience warmer conditions for their entire life. The potential for thermal plasticity of reproductive attributes due to development in elevated thermal conditions has been experimentally investigated in just a few species, all of them terrestrial insects [12], [39]–[40]. In the only other study to explore the potential for acclimation to temperatures above the present-day average, Zeh *et al.* [12] found no ability for a tropical pseudoscorpion to acclimate reproduction to a temperature increase of 3.5°C. Similarly, our fish reared at +3.0°C conditions did not show evidence of improvement in reproductive attributes, suggesting potential thermal limits to reproductive acclimation. Newly hatched fish from +3.0°C parents were smaller and lighter at hatching than +0.0°C offspring (as was found in the wild generation; [25]), possibly due to egg size being reduced in elevated temperature conditions (both +1.5 and +3.0°C), but there was no difference in the physical condition or amount of yolk provided to juveniles. The improvement in one offspring attribute (yolk area) when compared to the wild F_0_ generation, suggests that further generations held at +3.0°C may lead to improvements in other reproductive and offspring attributes.

There was no correlation between metabolic attributes and reproductive characteristics, suggesting that the capacity for oxygen supply and delivery was not responsible for differences in reproduction. In the +1.5°C treatment, a previous study [29] found no evidence of acclimation of aerobic scope in adults (i.e. aerobic scope exhibited the same decline in +1.5°C and +0.0°C fish), but in this study there were clear improvements in some aspects of reproductive ability with no apparent loss to physical condition. In the +3.0°C treatment group, while there was no evidence for improvement of aerobic scope with developmental acclimation, there was a partial compensation of resting metabolic rate [28]–[29], but we saw no improvement of reproductive output in this study. Additionally, the finding that F_1_ adults from the +3.0°C treatment had a lower resting metabolic rate [29] and higher HSI, than the +1.5°C adults, confirms that the lower reproductive output of +3.0°C pairs was not an effect of energy limitation. These results indicate that reproductive pathways are independently affected by water temperature and that aerobic performance will not be a good indicator of population persistence for all species [41]–[42]. It is possible that the lack of relationship between the metabolic and reproductive characteristics for this species is related to the mismatch between the thermal optimum for reproductive hormones and the optimum for metabolic enzymes. This highlights that to fully understand the potential for species to cope with future ocean warming we must examine a range of attributes that contribute to fitness and their potential to acclimate [43].

The observed enhancement of reproductive ability in the +1.5°C fish could be due to adjustments to the thermal sensitivity and optimal temperature range of the endocrine system. This may have occurred through changes to the thermal sensitivity of proteins and steroid hormones involved in reproduction [4], [23]. However, the production of smaller eggs by the +1.5°C pairs suggests that full compensation of the endocrine system did not occur within only one generation and perhaps the optimal range of activity for proteins and steroid hormones was increased, but not by the full amount. One potential mechanism by which smaller eggs could occur is through a reduced level of estradiol and vitellogenin in the plasma, which is known to correspond with reductions in the size of eggs produced [44]–[45]. While the exact mechanism underlying the response in the present study is unknown, there is clear evidence of improvement in reproductive performance when fish develop under +1.5°C elevated temperature conditions their entire life.

Depending on the selectivity of mortality agents, juveniles from the elevated temperature treatments could experience poorer survival. If mortality is size selective (towards the loss of small individuals) as is often found [46], it would be expected that F_2_ offspring produced by pairs of both elevated temperature treatments would suffer a higher mortality than offspring from +0.0°C parents. However, offspring from all parental treatments were in similar body condition and thus no differences in mortality would be expected if body attributes are the principle determinant of early mortality [47]–[48]. Importantly, the smaller newly hatched offspring produced by parents in elevated temperatures were able to compensate for their reduced size by 15 days post-hatching, possibly due to their enhanced metabolic attributes compared to the +0.0°C [32]. This accelerated growth is similar to the compensatory growth in the early life stages of marine fish found after periods of food limitation [49]–[51] and can come at a cost later in life [49], [52]–[53]. Nevertheless, compensatory growth could help overcome the detrimental effects of size-selective mortality in juveniles. Whether accelerated growth is maintained until later in life, or if there are costs associated with this period of fast growth, remains to be seen. It is possible that if juveniles had been reared with limited food availability then differences between temperature treatments may have been maintained [51]. However, the large yolk reserves at all temperatures (present study) and the enhanced metabolic attributes of +1.5 and +3.0°C progeny [32] may have allowed compensatory growth of juveniles regardless of rearing conditions.

Reproductive acclimation by *A. polyacanthus* to a 1.5°C increase may not be entirely surprising as populations of this species already reside in locations that experience this temperature during summer (e.g. Far Northern GBR and PNG), however those populations have potentially adapted (through natural selection) to the relatively warmer conditions over many generations. The present research establishes that within one generation improvements in reproductive ability are possible in response to a 1.5°C increase in water temperature. Perhaps more generations are required for acclimation of reproduction to greater temperature increases, or genetic adaptation may be required. This study highlights the need for longer-term experimental studies to better understand the effects of rising temperatures on fitness related traits and thus the potential for species to cope with projected climate change.
